# Multilevel perspectives on the implementation of the collaborative care model for depression and anxiety in primary care

**DOI:** 10.1186/s12888-024-05930-w

**Published:** 2024-07-22

**Authors:** Avram Kordon, Allison J. Carroll, Emily Fu, Lisa J. Rosenthal, Jeffrey T. Rado, Neil Jordan, C. Hendricks Brown, Justin D. Smith

**Affiliations:** 1 Northwestern University Feinberg School of Medicine, Chicago, IL USA; 2 Department of Psychiatry and Behavioral Sciences, Northwestern University Feinberg School of Medicine, Chicago, IL USA; 3 Center of Innovation for Complex Chronic Healthcare, Hines VA Hospital, Hines, IL USA; 4 Department of Population Health Sciences, Spencer Fox Eccles School of Medicine, University of Utah, Salt Lake City, UT USA

**Keywords:** Collaborative care, Implementation science, Primary care, Behavioral health, Qualitative research, Consolidated Framework for Implementation Research (CFIR)

## Abstract

**Background:**

The Collaborative Care Model (CoCM) is an evidence-based mental health treatment in primary care. A greater understanding of the determinants of successful CoCM implementation, particularly the characteristics of multi-level implementers, is needed.

**Methods:**

This study was a process evaluation of the Collaborative Behavioral Health Program (CBHP) study (NCT04321876) in which CoCM was implemented in 11 primary care practices. CBHP implementation included screening for depression and anxiety, referral to CBHP, and treatment with behavioral care managers (BCMs). Interviews were conducted 4- and 15-months post-implementation with BCMs, practice managers, and practice champions (primary care clinicians). We used framework-guided rapid qualitative analysis with the Consolidated Framework for Implementation Research, Version 2.0, focused on the Individuals domain, to analyze response data. These data represented the roles of Mid-Level Leaders (practice managers), Implementation Team Members (clinicians, support staff), Innovation Deliverers (BCMs), and Innovation Recipients (primary care/CBHP patients) and their characteristics (i.e., Need, Capability, Opportunity, Motivation).

**Results:**

Mid-level leaders (practice managers) were enthusiastic about CBHP (Motivation), appreciated integrating mental health services into primary care (Need), and had time to assist clinicians (Opportunity). Although CBHP lessened the burden for implementation team members (clinicians, staff; Need), some were hesitant to reallocate patient care (Motivation). Innovation deliverers (BCMs) were eager to deliver CBHP (Motivation) and confident in assisting patients (Capability); their opportunity to deliver CBHP could be limited by clinician referrals (Opportunity). Although CBHP alleviated barriers for innovation recipients (patients; Need), it was difficult to secure services for those with severe conditions (Capability) and certain insurance types (Opportunity).

**Conclusions:**

Overall, respondents favored sustaining CoCM and highlighted the positive impacts on the practice, health care team, and patients. Participants emphasized the benefits of integrating mental health services into primary care and how CBHP lessened the burden on clinicians while providing patients with comprehensive care. Barriers to CBHP implementation included ensuring appropriate patient referrals, providing treatment for patients with higher-level needs, and incentivizing clinician engagement. Future CoCM implementation should include strategies focused on education and training, encouraging clinician buy-in, and preparing referral paths for patients with more severe conditions or diverse needs.

**Trial registration:**

ClinicalTrials.gov(NCT04321876). Registered: March 25,2020. Retrospectively registered.

## Background

Depression and anxiety are leading causes of disability and disease burden worldwide. The subsequent health effects of depression and anxiety are profound, and comorbidities with other chronic illnesses such as arthritis, diabetes, and cardiovascular disease are common [1–5]. Many patients present to primary care for diagnosis and treatment of mental health conditions, and primary care clinicians are increasingly involved in the provision of mental health care [6–8]. Unfortunately, only a fraction of patients is properly identified and treated due to the time constraints of visits, under-detection of symptoms, and difficulty accessing psychiatrists [9–12].

The Collaborative Care Model (CoCM) is an integrated, evidence-based approach to treating mental health conditions such as depression and anxiety. The Advancing Integrated Mental Health Solutions (AIMS) Center at University of Washington, a leader in the design and application of CoCM, describes three key team members: primary care clinicians, psychiatric consultants, and behavioral care managers (BCMs) [13]. Primary care clinicians refer patients to the CoCM program. The psychiatric consultant supervises the BCM to develop treatment plans, recommend appropriate treatment modifications, and consult as needed. BCMs, typically master’s-level social workers, assess the patient, develop a treatment plan with the psychiatric consultant, communicate this plan to the patient’s clinician, and provide follow-up care to the patient. In previous studies, CoCM significantly decreased depressive symptoms, diminished time to symptom remission, and lowered risk for cardiovascular disease while improving treatment adherence and remaining cost-effective [14–21]. Although substantial evidence demonstrates the clinical benefits of CoCM, there is a dearth of research regarding its implementation and even less about its sustainment [22–25].

In clinical settings, successful implementation relies heavily on the capabilities and motivations of individuals who deliver the innovation and those beyond the team who provide support [26]. However, team membership and effectiveness are often overlooked and inadequately outlined in implementation frameworks [27]. In 2022, the Consolidated Framework for Implementation Research (CFIR) domains and constructs were updated in accordance with experienced user feedback [28]. The Individuals domain, in particular, was expanded to capture the nuances of individuals’ roles and characteristics in the success or failure of implementation.

The purpose of this study was to use the CFIR 2.0 Individuals domain to assess how implementation roles and characteristics influenced CoCM implementation. Within the context of a pragmatic implementation trial of CoCM, the analysis of multi-level perspectives provides both an expansion of the understanding of the implementation of CoCM and a thorough investigation of how individuals influence both implementation and sustainability. Interviews conducted with primary care clinicians, BCMs, and practice managers allowed us to apply the updated CFIR determinants framework to assess the complexities of individuals and teams in implementing and sustaining CoCM.

## Methods

### Study overview

This study was a process evaluation of the Collaborative Behavioral Health Program (CBHP) study (NCT04321876). The comprehensive protocol for this study is available elsewhere [29]. Briefly, this was a type 2 hybrid effectiveness-implementation study of CoCM using a randomized roll-out trial design conducted in 11 primary care practices in Northwestern Medicine (Chicago, Illinois) from 9/1/2018 to 1/31/2023. The nearly 120,000 primary care patients served by these practices were 66% white race, 8% Hispanic/Latino ethnicity, 59% female, and 22% ≥65 years of age; 20% had Medicare and 4% had Medicaid insurance coverage.

CBHP design and procedures were aligned with the AIMS Center model for CoCM [13]. Briefly, support staff screened primary care patients for depression using the PHQ-2 (with elevated scores followed-up with the PHQ-9) and anxiety using the GAD-7. Patients with scores ≥ 10 on the PHQ-9 and/or GAD-7 were eligible for referral to CBHP. The primary care clinician reviewed the PHQ-9 with the patient and introduced CBHP. If the patient was interested, the primary care clinician placed a referral to the BCM electronically or via warm handoff. The BCM conducted an initial assessment with the patient, met with the consulting psychiatrist to discuss assessments, and then communicated recommendations to the patient’s primary care clinician, who placed the medication prescription (per their discretion). The BCM called the patient every two weeks to reassess symptoms, met with the consulting psychiatrist weekly, and communicated medication changes or adjustments to the prescribing primary care clinician. If, through the initial assessment or follow-up calls, the BCM and consulting psychiatrist determined the patient had needs that exceeded the scope of CBHP, referrals were provided to connect the patient to adequate care.

Implementation strategies included leadership engagement, practice champions (an existing primary care clinician in each practice who advocated for CBHP to their colleagues), education and training, technical assistance, audit and feedback, and community feedback. Approximately 1–2 months before each practice’s CBHP implementation, a training session took place for primary care clinicians and support staff during a regularly scheduled meeting. Trainings were led by the practice’s assigned consulting psychiatrist and attended by the BCM as a first introduction. Training content included an introduction to the CoCM, an explanation of the new clinicians (i.e., BCM, consulting psychiatrist), each team member’s role, and procedures for screening, documentation, referrals, and billing. Each clinic had its own individual BCM and consulting psychiatrist.

This process evaluation is focused on the perspectives of various implementers’ involved in CBHP implementation. The main outcomes of the trial will be reported elsewhere. All research procedures were approved by the Northwestern University Institutional Review Board.

### Participants

We recruited practice champions, BCMs, and practice managers who worked in the 11 practices that implemented CBHP via direct outreach. No other inclusion or exclusion criteria were applied. We interviewed 35 participants at 4 months to assess early implementation (12 practice managers, 11 practice champions, and 12 BCMs) and 33 participants at 15 months to assess sustainment (11 practice managers, 11 practice champions, and 11 BCMs). Participants provided verbal informed consent and received a $10 gift card for their time. Practice managers were predominantly female and had a graduate degree (e.g., Masters, MBA). Practice champions were predominantly female and had a medical school education (MDs). BCMs were predominantly female licensed clinical social workers (LCSWs). Information concerning age, race, and ethnicity was not collected.

### Interviews

Semi-structured, 30-minute qualitative interviews were conducted at 4- and 15-months after the program launched in each practice, during the early implementation and sustainment phases, respectively. Interviews were tailored to each participant role. The interview guides were informed by the CFIR 1.0 (the available version at the time of study design) to identify barriers and facilitators to CBHP implementation, program effectiveness, and care coordination across departments. Sample questions that explored the influence of individual characteristics include: “What were your day-to-day responsibilities? What is your understanding of CBHP? What has been your personal experience with integrating behavioral health services in primary care?” Interviews were conducted by EF, a clinical psychology predoctoral trainee, and AJC, a faculty member, with extensive experience and training in qualitative data collection methods. Interviews were conducted either in-person or virtually, and audio responses were recorded and transcribed for analysis.

### Qualitative analysis

We used framework-guided rapid analysis based on organized themes [30]. We used the CFIR 2.0 Construct Mapping document to convert the interview guide, developed using CFIR 1.0 constructs, to inform the analysis template using CFIR 2.0 domains and constructs [30, 31]. Transcripts of recordings were analyzed by EF and AJC who have training in framework-guided rapid analysis and have employed these methods in previous studies [32, 33]. After double-coding the first 3 transcripts together (one per participant group), the remaining transcripts were coded individually. Any discrepancies that arose were discussed among team members along with the study principal investigator, JDS, and a solution was derived via collaborative consensus. Templated summaries were used to consolidate response data [30].

Per the CFIR 2.0 Individuals domain [28], interview responses were first evaluated by the project roles that were represented (see Table 1 for the coding of response data to capture participant perspectives). The Individual role codes included *Mid-Level Leaders* (i.e., practice managers), *Implementation Team Members* (i.e., primary care team members [clinicians, practice champions, support staff, administrative staff]), *Innovation Deliverers* (i.e., BCMs), and *Innovation Recipients* (i.e., primary care patients and CBHP participants). Practice managers were categorized as Mid-Level Leaders because of their role in overseeing CBHP implementation. Primary care team members were categorized as Implementation Team Members because of their role in supporting CBHP implementation via referring patients to CBHP and collaborating with those who directly implemented the innovation. BCMs were categorized as Innovation Deliverers because of their role in directly providing patient treatment after CBHP referral. Consulting psychiatrists would also be considered *Innovation Deliverers,* but they were not included in the process evaluation for this study. Also, because this was a pragmatic implementation trial, patient perspectives were not directly queried and, as such, the results capture other participants’ perspectives of the patients’ characteristics.

**Table 1:** Coding of Collaborative Behavioral Health Program (CBHP) participant by CFIR role and interview status

Position	CFIR role in CBHP implementation	Interview Participant
Practice Manager	Mid-Level Leader	Yes
Practice Champion	Implementation team member	Yes
Primary Care Clinician	Implementation team member	No
Practice Support and Administrative Staff	Implementation team member	No
Behavioral Care Manager	Innovation Deliverers	Yes
Consulting Psychiatrist	Innovation Deliverers	No
Primary Care and CBHP Patients	Innovation Recipients	No

Second, interview responses were coded according to the role’s project characteristics [28]. *Need* refers to how an innovation increases well-being by addressing inadequacies in current care. *Capability* refers to an individual’s ability to successfully carry out responsibilities with confidence. *Opportunity* refers to aspects outside of an individual’s control that influence behavior and the success of implementation. *Motivation* refers to an individual’s level of interest in carrying out their responsibilities. The other CFIR domains were also queried and will be reported elsewhere; the purpose of this report is to provide an in-depth exploration of the Individuals domain.

## Results

The results of the qualitative coding are presented by the CFIR 2.0 project roles that were represented in the interview responses (i.e., Mid-Level Leaders, Implementation Team Members, Innovation Deliverers, Innovation Recipients). Notably, interview responses are aggregated such that the results represent the direct views of interview participants as well as interviewees’ perspectives of other roles (see Table 1). The results are further subdivided into characteristics (i.e., Need, Capability, Opportunity, Motivation), defined above. A summary of the results is presented in Table 2.

**Table 2:** Collaborative Behavioral Health Program (CBHP) response data from 4-month and 15-month interviews, by project role and characteristic

Role	Characteristic			
	**Need**	**Capability**	**Opportunity**	**Motivation**
**Mid-Level Leaders** (Practice Managers)	• More integration of mental health services into primary care practices • Better interdepartmental communication between primary care and psychiatry	[No data]	• Assisted with appointment scheduling • Facilitated communication within the team • Limited scope to connect the team with external individuals	• Broad enthusiastic engagement • Strong team member communication • Supportive environment
**Implementation Team Members** (Primary Care Team Members)	• Decreased clinician burden	• Successfully referred patients to CBHP • Unable to adequately treat and refer patients with complex needs • Lack of familiarity with CBHP requirements and scope among clinicians	• Clinicians had the greatest influence on whether CBHP was utilized • Implementing CBHP could be time-consuming • Some with limited engagement in depression screening • Interactions with other team members could be limited to EHR-based communication • More mental health information and resources desired • Support staff facilitated communication and screenings; less responsibility in CBHP itself	• Excitement to have CBHP as a resource • Lack of treatment options for patients with complex mental health needs • Some hesitancy to give up some autonomy over patient treatment • Increased clinician confidence to treat patients with symptom exacerbations
**Innovation Deliverers** (Behavioral Care Managers)	• Frequent and consistent referrals received from clinicians; differed by practice	• Confidence in ability to assist patients and coordinate care • Confidence of other team members in the BCMs’ abilities • Challenging to handle inappropriate CBHP referrals	• Caseload depended on clinician referrals • In-person (vs. virtual) interactions facilitated team communication • Difficulty managing patients who did not follow-up	• Eager to implement CBHP
**Innovation Recipients** (Primary Care Patients and CBHP Participants)	• Long wait times for psychiatry and other mental health resources • Mental health needs extend beyond moderate depression and anxiety • CBHP provided timely and effective care as well as case management • Identifying more patients who would benefit from services via depression screening	• Depression may interfere with ability to engage in treatment	• Regular, frequent follow-up provided structure and support • Insurance coverage can limit patient opportunity to participate in CBHP	• Ambivalence toward psychiatric medications • Hesitancy about receiving treatment from individuals other than their primary care clinician • Frustration with slow improvements

### Mid-level leaders

#### Need

At both time points, all practice managers emphasized the vital importance of how the CBHP workflow expanded the resources providers could draw upon for mental health care and appreciated BCMs’ role in bridging this connection. One participant stated, “Absolutely. It’s very necessary to have someone onsite.” Regarding the sustainability of CBHP, all practice managers believed it should continue, e.g., “Definitely. I can’t see our practice without them.” Several practice managers also illustrated the potential CBHP had to improve connections and referral pathways between primary care and psychiatry. One participant referred to CBHP as an “all-in-one model.”

#### Capability

None of the responses analyzed reflected Capability of Mid-Level Leaders.

#### Opportunity

Practice managers explained they were able to assist their own clinicians with scheduling appointments and facilitating communication between team members. However, they were unable to address concerns about communication with individuals outside of the team (e.g., psychiatry department) due to the psychiatrists’ demanding schedules and limited availability, which limited their opportunity to achieve interdepartmental collaboration.

#### Motivation

During both the 4- and 15-month interviews, practice managers reported hearing positive feedback about CBHP team members’ communication and feelings of support. At the 4-month interview, BCMs highlighted how practice managers were available and valuable. As one BCM related, practice managers were “very supportive… because they wanted the program.” Practice managers expressed broad enthusiastic engagement when interviewed during both phases. In response to whether the program should continue, one practice manager responded, “1,000%. If anything, I want more.” At 15 months, practice managers continued to support CBHP and its sustainability.

### Implementation team members

#### Need

In the 4-month interviews, participants in all roles expressed how CBHP helped lessen the burden the health care team experienced. Practice champions reported CBHP made clinicians feel supported and reduced their workload (e.g., responding to patient messages). This feeling of support persisted into the 15-month interviews. As one BCM stated, “Providers have said they feel like a weight has been lifted.” Practice champions also described how clinicians praised the resources provided by CBHP, which allowed for an expansion of treatment options and more consistent management of mental health needs. Likewise, BCMs and practice managers related CBHP allowed clinicians to grow their knowledge of mental health disorders and treatment. As one practice manager stated, “It’s been a good resource for [clinicians] to ask questions.” At 15-months, one practice champion asserted, “It would be chaotic if it wasn’t here anymore.”

#### Capability

Practice champions described that, despite CBHP, clinicians did not feel they had the ability to provide adequate referrals for patients with urgent mental health needs, and they commented on the difficulty clinicians experienced in connecting patients with psychiatrists. For patients with less severe needs, BCMs largely agreed clinicians appropriately referred patients to CBHP and provided the necessary information. At 4-months, several participants reported clinicians were not always familiar with CBHP’s eligibility criteria and may have benefitted from re-education and training.

#### Opportunity

In general, BCMs felt primary care clinicians had the greatest influence on whether CBHP was utilized, as clinicians were responsible for referring to CBHP. Some BCMs and clinicians also expressed during both phases that CBHP could be time-consuming. Namely, practice champions stated conducting screenings and explaining the program took away time from the visit, and it was easier to simply refer a patient without screening or explanation. Moreover, participants indicated that while some or even most clinicians adhered to screening guidelines, not all clinicians did so, which limited the probability they would identify and refer an eligible patient to CBHP. Additionally, while conversations between BCMs and clinicians occurred in-person at several locations, after the onset of the COVID-19 pandemic, much of the communication shifted to the EHR. At 15-months, BCMs also described how clinicians were frustrated when patients could not be seen sooner by other services (e.g., psychiatry department, community-based psychiatrists). Regarding support staff, some BCMs expressed that nurses and medical assistants were helpful in facilitating communication. However, the prevailing theme was the staff were not significantly involved in CBHP and, rather, their responsibility was to conduct screenings.

#### Motivation

During the 4-month interview, practice managers emphasized CBHP was particularly helpful for clinicians who were treating patients during symptom exacerbations. This approach allowed clinicians to feel more confident, knowing a patient’s needs would be met. At both interview points, most practice champions expressed clinicians had an enthusiastic interest in the program. Practice managers and BCMs also stated they felt many clinicians were excited about CBHP and enjoyed having the program as a patient resource. As one BCM stated during the 4-month interview, “We’ve gotten so many referrals.” Further underlining this support, one practice manager asserted, “Our staff don’t complain about anything extra that they have to do.”

However, some practice champions and practice managers expressed that they observed hesitancy from other clinicians. Several practice champions reported there were ways that CBHP did not adequately address clinicians’ needs during implementation and sustainment. A repeated wish described by practice champions was that CBHP be expanded to treat more complex psychiatric disorders. Various participants also detailed how several clinicians did not utilize the program because they preferred to treat symptoms on their own. During the 4-month interview, one practice champion explained how some clinicians were against writing prescriptions and “remotely treating” patients they were not fully counselling. In a 15-month interview, one practice champion stated, “physicians like their autonomy.”

### Innovation deliverers

#### Need

Several BCMs expressed CBHP provided them with a means to support patients who needed mental health care. One BCM remarked, however, that they also needed implementation team members to refer their patients and provide CBHP as an option.

#### Capability

BCMs generally expressed confidence in their ability to assist patients and coordinate care between the primary care clinicians and psychiatrists. BCMs also reported they often conducted depression and anxiety screening themselves when the clinicians and staff were unable to do so. Practice champions conveyed that clinicians broadly valued BCMs’ expertise and capacity to follow-up with patients independent of clinicians. Practice managers also expressed clinicians felt positively about BCMs’ ability to communicate and especially valued how BCMs could connect directly with psychiatrists. One limitation of the innovation itself (CBHP) described by BCMs during the 4-month interview was referrals for behaviorally complex diagnoses that were beyond the scope of CBHP per the AIMS model and thus beyond BCMs’ scope of practice (e.g., substance use).

#### Opportunity

Several BCMs at the 4-month interview emphasized that to fulfill their role, clinicians first needed to refer suitable patients. Additionally, during the 4-month interview, several BCMs explained they were better able to perform some of their responsibilities, such as communicating with clinicians, when working in-person as opposed to online (e.g., via email or EHR messages), a common effect of the COVID-19 pandemic. One BCM also explained their opportunity to provide treatment was limited if patients did not follow-up with messages. Finally, practice champions espoused the value of the BCM component of CBHP. As one practice champion stated during the 4-month interview, “She’s… extremely accessible and available, and she’s just been a really great resource.”

#### Motivation

In general, BCMs were eager to work within the CoCM model to implement CBHP and ensure proper patient care.

### Innovation recipients

#### Need

Practice managers, BCMs, and practice champions discussed the many mental health treatment needs of their patients, including the inaccessibility of psychiatry referrals and long wait times, stigma around talking about mental health issues, and suffering from mental health concerns. At both the 4- and 15-month interviews, participants felt CBHP helped to partially alleviate these barriers and provide holistic care to patients. As one BCM explained during the 4-month interview, “It’s not just addressing symptoms. We can spend a lot more time with them… because their [clinician] visits are so much shorter.” Likewise, many practice managers felt CBHP provided an outlet for more regular patient follow-up, enhanced patient participation in mental health care, and appeared to improve patients’ health more quickly than standard practice. Practice managers especially felt depression and anxiety screening was an important element of CBHP for patients. As one practice manager stated during the 15-month interview, “It’s a great idea, we weren’t capturing these patients before.” Nonetheless, participants across all project roles indicated they wished the resources provided by CBHP would be expanded to encompass needs outside of common mood disorders (e.g., depression and anxiety) such as substance use disorder, ADHD, and severe mental illness.

#### Capability

Some BCMs at the 4-month interview reported it can be difficult for patients with depression and anxiety to engage in treatment and make progress. Nevertheless, BCMs also emphasized how their connections and communications with psychiatric consultants allowed them to serve as a bridge for patients to engage in psychiatric care, thus reaching and engaging more patients overall.

#### Opportunity

During the 4-month interview, one BCM mentioned CBHP gave patients time and structure to reflect on and benefit from their treatment due to the frequency of follow-up appointments. A barrier reported by BCMs at both interview points was that insurance coverage prevented some patients from utilizing CBHP.

#### Motivation

Common barriers to patient commitment to CBHP expressed by BCMs during the 4-month interview were an ambivalence towards mental health treatment and a hesitancy to begin psychiatric medication. One practice manager commented that it was difficult to motivate patients to follow through with referrals. During the 15-month interview, one practice manager and BCM said some patients wished improvement would happen more quickly, and providers needed to manage patient expectations about the speed of recovery. Participants also expressed some patients come to primary care expecting their clinician to directly manage their care and, thus, were discouraged when connected to BCMs; this perhaps indicates a lack of clear communication about the team-based nature of CBHP.

## Discussion

The aim of this secondary qualitative analysis of a type 2 hybrid effectiveness-implementation study of CoCM was to explore determinants of CoCM implementation and sustainment, guided by the updated CFIR 2.0’s Individuals domain. Overall, participants were in favor of CoCM and highlighted the positive impacts of CBHP regarding the practice, health care team, and patients. Participants emphasized the benefits of integrating mental health services into primary care, and participants across all project roles highlighted how CBHP lessened the burden on clinicians while providing patients with comprehensive care. Iterative implementation changes resulting from these interviews included refining education and training materials, instituting re-education sessions, and employing more frequent feedback regarding CBHP patient outcomes.

Overall, CBHP helped to overcome obstacles patients and providers face in engaging and treating primary care patients with depression and anxiety. Across all project roles, participants detailed the substantial barriers patients typically face to obtain sufficient mental health care, including long wait times for psychiatry appointments, stigma around mental health concerns, and time constraints of primary care visits, all of which are long-standing problems in the U.S. [11, 34, 35]. Many participants articulated how CBHP helped alleviate these barriers for their patients. BCMs and practice managers stated CBHP allowed for more holistic patient care, and practice champions conveyed CBHP helped to improve patients’ health more quickly than standard practices by providing an outlet for more regular follow-up. The diverse benefits of CoCM have been published elsewhere [14, 15, 17–21], with one study observing that patients who participated in CoCM achieved remission an average of 18 months sooner than those who received usual care [16]. Participants also emphasized that primary care clinicians are extremely busy, and CBHP lessened clinician burden and provided the support needed to address patients’ mental health needs. Alternatively, there were some clinicians who did not agree CBHP addressed all their patient needs, such as the treatment of patients with more complex disorders, or because they wanted to be more hands-on with their patients. Notably, however, the only patient inclusion criterion for CoCM is mild to moderate depression or anxiety, and thus this program is not intended to manage severe or complex cases. Future CoCM implementation may benefit from using strategies to increase clinician buy-in and prepare appropriate referral paths for patients with more severe or diverse disorders (e.g., ADHD, substance use disorders) not addressed by CBHP.

Interpersonal competence to carry out individual responsibilities is a vital part of working on an interdisciplinary care team. CBHP participants across all project roles felt capable, and described others on their team as capable, in their abilities to ensure satisfactory patient care. Practice managers received positive feedback about team member communication, clinicians successfully referred patients to BCMs, and BCMs felt equipped to assess and treat patients and coordinate care for those whose mental health problems were beyond the scope of CBHP. Nonetheless, at times practice champions expressed it was difficult for clinicians to manage patients who were not eligible for CBHP due to severe mental health conditions or urgent mental health needs, a common limitation of CoCM programs [36]. Some participants also stated some clinicians were unfamiliar with CBHP program criteria and goals and expressed frustration with its limitations. It is vital that all members of health care teams have the means to fulfill any role they take on through appropriate education, training, and re-training throughout implementation [37].

Most individuals involved in the implementation of CBHP felt they had opportunity to fulfill their roles, though there were notable barriers. First, BCMs noted they relied on clinicians referring suitable patients to successfully do their job. Strategies to increase patient reach, such as panel management and proactive screening measures, may further overcome this barrier and decrease clinician burden [38]. Second, while many team members described favorable interactions and teamwork with clinicians and staff, some BCMs desired a more cohesive team atmosphere, consistent with prior research indicating social workers highly value interpersonal relationships [39]. The dynamics of a care team influence individuals’ power and opportunity to fulfill their assigned roles [40]. Third, some patients lacked insurance coverage for CBHP. Based on the positive effects CoCM has on healthcare delivery and outcomes, it is imperative we continue to promote policy changes that would allow payors to include CoCM as a fully covered and reimbursable service [14–21, 41, 42].

Finally, individuals were motivated to fulfill their roles, and most participants were enthusiastically invested in its implementation and sustainment. For clinicians, a significant motivating factor was BCMs’ ability to handle treatment modifications appropriately. Given the extensive process of follow-up appointments for informed dosage adjustments for psychiatric medications, it can be time-intensive to continually monitor patient needs and react quickly to flare-ups [43]. Thus, CBHP bolstered clinicians’ confidence in treating patients who experienced exacerbations of symptoms. From the patient perspective, some participants reported hesitancies and barriers (e.g., ambivalence toward psychiatric medications), on which we have previously reported [44]. Some clinicians also preferred to be the primary provider for their patients’ mental health needs and were less likely to refer to CBHP. Because of the dynamic interplay between clinician behavior and patient engagement, strategies to promote clinician buy-in, such as outlining clear communication plans and cultivating team synergy, would likely support CoCM adoption [45–47].

Applying CFIR 2.0 allowed us to fully appreciate how the needs, capabilities, opportunities, and motivations of multi-level implementors determined the success of CoCM implementation. With the development and expansion of CFIR from Version 1.0 to 2.0, the restructuring of the Individuals domain was designed to better understand the unique effects of individual roles and characteristics [28]. Individuals are active participants in the implementation of any innovation, and the complex relationship they maintain with the innovation and the organization within which it is implemented greatly affects the success of that innovation. We found the focus on individual project roles and characteristics provided multi-level insights into how CoCM was incorporated into practices along with areas for additional implementation strategies.

Limitations of this study should be noted. First, only BCMs, practice managers, and practice champions were interviewed. Including other perspectives, such as psychiatric consultants, non-practice champion primary care clinicians, support staff and administrators, high-level leaders, and patients, would likely provide further insights and a more holistic perspective. Notably, insights from patients who failed to enroll in CBHP are published elsewhere [44]. Second, while we conducted an in-depth analysis of the CFIR 2.0 Individuals domain, a full evaluation of all five domains (Innovation, Outer Setting, Inner Setting, Implementation Process) would also lend valuable information.

## Conclusions

CoCM is an effective intervention for depression and anxiety and reduces the burden on clinicians and support staff. Understanding the individual roles and characteristics that influence implementation is a key domain critical for implementation engagement and adoption. This study provides an example of an in-depth exploration of the CFIR 2.0 Individuals domain and identifies key determinants to be addressed by effective strategies for future CoCM implementation and sustainment.

## Data Availability

The datasets used and analyzed during the current study are available from the corresponding author on reasonable request.

## Abbreviations

CoCM: Collaborative Care Model for Depression
AIMS: Advancing Integrated Mental Health Solutions
BCM: Behavioral Care Manager
CFIR: Consolidated Framework for Implementation Research
CBHP: Collaborative Behavioral Health Program
PHQ-9: Patient Health Questionnaire
GAD-7: General Anxiety Disorder Questionnaire
